# c.98 + 3A>G and c.155 + 1G>T splice-site variants in the ABO*B.01 allele lead to weak antigen expression in the Chinese individuals

**DOI:** 10.3389/fimmu.2026.1739842

**Published:** 2026-04-22

**Authors:** Jingjing Zhang, Yanling Ying, Xiaozhen Hong, Faming Zhu

**Affiliations:** Blood Center of Zhejiang Province, Hangzhou, China

**Keywords:** ABO gene, antigen expression, exon skipping, function analysis, splice-site variant

## Abstract

**Background:**

Many variants within the *ABO* gene across multiple subtypes have been identified. These variants are located in the coding region, the erythroid-specific regulatory element region of intron 1, splice sites, and other non-coding regions. However, data regarding the functional analysis of these variants remain limited. In this study, we analyzed the function of two splice-site variants in ABO subtypes.

**Methods:**

ABO phenotyping was performed using conventional serology methods. The entire coding sequence of the *ABO* gene was characterized by polymerase chain reaction sequence-based typing, while haplotypes were determined using one-step long-range PCR combined with single-molecule real-time sequencing. The functional impact of splice-site variants was predicted using *in silico* tools and subsequently verified *in vitro* using a minigene splicing assay. Furthermore, stable cell lines expressing ABO cDNA with exon 2 or 3 deletions were established to evaluate their effects on antigen expression utilizing serology, flow cytometry, and glycosyltransferase activity assays.

**Results:**

The c.98 + 3 heterozygous variants were identified in Bw and ABw individuals, while the c.155 + 1 heterozygous variant was identified in an ABw individual. In all three individuals, these splice-site variants could be assigned to the ABO*B.01 allele by haplotype analysis. These variants affected splicing as predicted by *in silico* analysis, resulting in exons 2 or 3 skipping, as demonstrated by minigene assays. B antigen expression was significantly weakened in cells transfected *in vitro* with ABO*B.01 cDNA containing deletions in exons 2 or 3, as analyzed by cytometry and serological methods. In addition, no enzymatic activity was detected in the supernatant of these transfected cells.

**Conclusions:**

The c.98 + 3A>G and c.155 + 1G>T variants within the *ABO* gene may induce aberrant mRNA splicing, leading to skipping of exons 2 or 3, and resulting in the formation of a nonfunctional glycosyltransferase.

## Introduction

The ABO blood group system plays an important role in transfusion medicine (1). It is also associated with the occurrence and progression of some diseases (2, 3). ABO incompatibility can trigger a severe hemolytic reaction in transfusion or may lead to the development of hemolytic disease in newborns (4, 5). The ABO blood group system is typically classified into A, B, O, and AB phenotypes; however, a small proportion of diverse ABO subtypes exists in various populations (6, 7). A deeper understanding of the molecular mechanisms underlying the differential antigen expression of these ABO subtypes is crucial for their accurate identification and for improving transfusion safety (6–8).

The *ABO* gene spans more than 20 kb and consists of seven exons (28, 70, 57, 48, 36, 135, and 691 bp, respectively). It encodes the A glycosyltransferase (GTA) and/or B glycosyltransferase (GTB). GTA catalyzes the transfer of UDP-N-acetylgalactosamine to the H substrate, forming the A antigen, whereas GTB transfers UDP-galactose to the H substrate to form the B antigen. However, variants in the *ABO* gene can decrease the activity of GTA and/or GTB, leading to a weak or no A and/or B antigen expression in these individuals (9, 10). To date, over 400 ABO variants have been reported (11), including nearly 60 variants previously identified and submitted by our laboratory in earlier studies (12–14). These variants in the *ABO* gene arise from base substitution (15), insertion (16), deletion (17), and recombination events (18) in the coding sequence, as well as from base substitutions in the erythroid-specific regulatory element region of intron 1 (19), alterations at splice sites (20), changes in the ABO promoter (21), and modifications in upstream/downstream cis- or trans-regulatory elements (22).

RNA splicing is a common and critical biological process in eukaryotic gene expression. By recognizing sequences at exon-intron boundaries and splice sites in pre-mRNAs, the splicing machinery can generate different transcriptional variants (23). These mRNA splice variants lead to significant differences in the amino acid sequence of the encoded protein compared with that of the constitutive protein and result in varying degrees of functional alterations (23, 24). Some variants in the splice sites in the *ABO* gene have been identified. Six of these variants were identified in nine individuals in our previous study (20). However, the data on *in vitro* functional analysis of these variants are limited. Theoretically, such splice-site variants can generate a novel RNA splice event, affect glycosyltransferase activity, and give rise to distinct ABO subtypes. In this study, two intronic splice-site variants, c.98 + 3A>G and c.155 + 1G>T, were identified in ABO subtypes. The c.98 + 3A>G variant has been previously reported (25), while the c.155 + 1G>T variant represents a novel finding. Furthermore, for the first time, minigene technique and *in vitro* expression experiments were performed to elucidate the effects of these variants on glycosyltransferase activity.

## Methods

### Study specimens

Among blood donors at the Blood Center of Zhejiang Province, China, three probands were identified based on initial ABO typing that revealed weak expression of the B antigen or discordance between forward and reverse typing, requiring further analysis. Written informed consent was obtained from all participants. The project was approved by the Ethics Committee of the Blood Center of Zhejiang Province (approval number: 2020-010).

### Serological tests for the ABO blood group

A, B, and H antigens, as well as anti-A and anti-B antibodies, were detected using conventional serological methods according to our previous report (20). Both the antibody reagents (anti-A, anti-A1, anti-B, anti-AB, and anti-H) and the group A, B, and O red blood cells were procured from a commercial company (Shanghai Blood Bio-technology Co., Ltd, Shanghai, China).

### Sequencing analysis for the full exons of the *ABO* gene

Genomic DNA was extracted from the blood cells using a commercial MagDNA pure LC DNA isolation kit according to our previous report (20). Complete coding sequence (CDS) of the *ABO* gene was analyzed using the polymerase chain reaction sequence-based typing (PCR-SBT) technique (15, 20, 26). In brief, three pairs of primers were used to amplify all exons of the *ABO* gene and sequenced using the Sanger method. The reference sequence of the *ABO* gene was obtained from GenBank (NG_006669.2), and the ABO genotype was determined based on nucleotide polymorphisms.

### Analysis of the *ABO* gene haplotype

The haplotype was analyzed using one-step long-range PCR (LR-PCR) with the single-molecule real-time sequencing method according to our previous report (27). A one-step LR-PCR method was used to amplify the entire the *ABO* gene and then sequenced on the Sequel II platform (Pacific Biosciences, California).

### *In silico* prediction for splice-site variants

Splice-site prediction analysis was performed using Alamut™ Visual Plus software with four splice site prediction tools(SpliceSite Finder-like, MaxEntScan, NNSPLICE, and GeneSplicer) to predict the effects of these splice site variations (www.sophiagenetics.com/sophia-ddm-for-genomics/alamut-visual-plus/). Additionally, the state-of-the-art deep learning tool SpliceAI(version 1.3.1) (https://spliceailookup.broadinstitute.org/#) was used to assess variant effects, providing donor loss (Δ) scores, ranging from 0 to 1. Scores closer to 1 indicate a higher probability that the variant disrupts the canonical splice donor site.

### Analysis of RNA splicing of the variants using the minigene technique

#### Construct minigene reporter plasmid

According to the different exon-intron splicing variation sites of each specimen, a single minigene construct was generated by cloning three discontinuous sequences of the *ABO* gene, including exon 1, the test exon (exon 2 or 3, respectively), and exon 7, along with their flanking sequences(including the potential splicing sites) into the pcDNA3.1(+) vector (Invitrogen, Shanghai, China). The sequences were synthesized and cloned into the pcDNA3.1(+) vector by a commercial company (Genscript, Nanjing, China). All sequences are listed in **Supplementary Table 1**. To verify the accuracy of the sequence, the recombination plasmid DNA was sequenced using the BigDye^®^ Terminator v3.1 Cycle Sequencing Kits (Thermo Fisher Scientific, Shanghai, China) and ABI 3730 automated sequencer according to the manufacturer’s instructions. All sequence primers are listed in **Supplementary Table 2**. The nucleotide sequences were analyzed using the SeqScape v2.5 software (Thermo Fisher Scientific, Shanghai, China). Four different recombinant plasmids were constructed. Two of them were with c.98 + 3G and c.155 + 1T, respectively, while the corresponding control constructs contained the wild-type sequences at the respective splice sites (c.98 + 3A and c.155 + 1G).

#### Minigene reporter plasmid into K562 cells using electrotransfection

The recombinant plasmid was transfected into K562 cells using the electrotransfection method according to the manufacturer’s instructions. K562 cells were harvested after culture, washed in phosphate-buffered saline (PBS) three times, and resuspended at 1.5×10^7^ cells/mL for usage. In total, 30µg plasmid DNA was added to 200µL cell suspension and transferred to 0.2cm gap Bio-Rad electroporation cuvettes. A single pulse was delivered (GenePulser Xcell™ Electroporation System, Bio-Rad, Shanghai, China) at 1000 µF, 110V. Cells were immediately resuspended in 2mL Dulbecco’s Modified Eagle Medium (Thermo Fisher Scientific, Shanghai, China) supplemented with 10% fetal serum (Thermo Fisher Scientific, Shanghai, China) and incubated at 37°C (95% air and 5% CO_2_) for 48 h.

#### RT-PCR analysis for ABO cDNA in the K562 cells with minigene reporter plasmid

Total RNA was extracted from transfected cells using the QIAamp^®^ RNA Blood Mini Kit (Qiagen Company, Hilden, Germany) according to the manufacturer’s instructions. RNA was reverse transcribed into cDNA using the PrimeScritTM Reagent Kit (RR037A, Takara, Dalian, China). cDNA was then amplified using the primers and 2x TransStart^®^ FastPfu Fly PCR SuperMix kit (AS231, TransGen Biotech, Beijing, China), the sequences of forward and reverse primers were 5’ATGGCCGAGGTGTTGCGGAC 3’ and 5’TAATCCACCTCGCTGAGGAA 3’, located in exons 1 and 7, respectively. Amplions were separated by electrophoresis through an agarose gel, and the DNA size marker was used as a control (Takara, Dalian, China). The gel of each ABO cDNA band in K562 cells was cut and purified using QIAquick^®^ Gel Extraction Kit (Qiagen Company, Hilden, Germany), sequenced, and analyzed as described in the “Construct minigene reporter plasmid” section. Detailed protocols are provided in **Supplementary Methods**.

### Construct ABO cDNA cells without the expression of exons 2 or 3

#### Synthesis of ABO cDNA specific expression plasmid

The cDNA sequences of ABO*B.01 allele without exons 2 or 3 were synthesized and cloned into the pcDNA3.1(+) vector by a commercial company (Genscript, Nanjing, China). The cDNA of the ABO*B.01 allele (NM_020469.2) was also inserted into pcDNA3.1 (+) vector as a positive control, and only pcDNA3.1 (+) vector as negative control. To verify the accuracy of the sequence, recombination plasmid DNAs were sequenced using the BigDye^®^ Terminator v3.1 Cycle Sequencing Kits (Thermo Fisher Scientific, Shanghai, China) according to the manufacturer’s instructions. Three recombination plasmids (ABO*B.01 cDNA with exon 2 deletion, ABO*B.01 cDNA with exon 3 deletion, and ABO*B.01 cDNA) and the original pcDNA3.1 (+) vector were obtained for further study.

#### Cell transfection with ABO cDNA specific expression plasmid

HeLa cells (The Cell Bank of the Typical Culture Preservation Committee of the Chinese Academy of Sciences, Beijing, China) were cultured using conventional cell culture methods. Three specific expression recombination plasmids and the original pcDNA3.1(+) vector were transfected into HeLa cells with Lipofectamine™ 3000 Transfection Kit (Invitrogen, Shanghai, China), according to manufacturer’s instructions.

#### Screening of stable expression cells with specific ABO cDNA

After cell transfection, G418 was added to the transfected cells containing complete medium for culture. The optimal concentration of G418 of 1000 µg/mL was maintained. The cloned cells were cultured using the limited dilution method until a single cell was obtained. The culture system was gradually expanded until a certain number of stable monoclonal cells were obtained. Culture supernatants of the monoclonal cell were retained for further use. At least three independent monoclonal cell lines were isolated and expanded to ensure reproducibility of the results.

### Analysis of B antigen expression and glycosyltransferase activity of the transfected cells

#### Analysis of the B antigen by serological test

B antigen on the transfected cells was detected with anti-B antibody (Shanghai Blood Bio-technology, Shanghai, China) using a conventional serological method (20).

#### Analysis of the B antigen by flow cytometry

Typically, 2%-5% cell suspension was prepared, and PE fluorescent-labeled anti-B antibody (ARP, Massachusetts, USA) was added according to the manufacturer’s instructions. Upon mixing, the cell suspension and antibody were reacted in the dark for 30min at room temperature, then 500 μL PBS was added to mix and immediately detected on the Flow cytometer (BD, New Jersey, USA). 10, 000 cells were obtained for fluorescence analysis by BD FACSuite software. Cell surface antigen expression was calculated according to the mean fluorescence intensity. Detailed protocols are provided in **Supplementary Methods**.

#### B glycosyltransferase activity assay of the transfected cells

The GTB activity in the culture supernatants was assayed according to our previously described method (28). Detailed protocols are provided in **Supplementary Methods**.

### Quantitative real-time PCR

Total RNA extraction and cDNA synthesis were performed as described above in the section “RT-PCR analysis for ABO cDNA in the K562 cells with minigene reporter plasmid”. mRNA expression was quantified by TaqMan probe-based qRT-PCR using a duplex assay that co-amplified the target gene and the endogenous control (GAPDH) in the same reaction. Relative expression levels were calculated using the 2^(-ΔΔCt) method. Data are presented as the mean ± standard deviation (SD) from three independent experiments. Detailed protocols are provided in **Supplementary Methods**.

## Results

### Serological results of the probands

Three Chinese blood donors were studied. The agglutination reaction states of these individuals’ RBCs with anti-A, anti-B, anti-AB, anti-A1, anti-H antibodies, and serum with known A, B, and O group RBCs are listed in **Table 1**. RBCs of all three individuals exhibited 3+ or 4+ strength agglutination with anti-H antibody. ID 1 donor demonstrated mixed-field agglutination with anti-B antibody. These individuals were categorized into the Bw subtype based on their serological characteristics.

**Table 1:** Results of serological grouping and genotype analysis for the probands with ABO subtypes.

Forward typing test						Reverse typing test					
ID	Anti-A	Anti-B	Anti-AB	Anti-A1	Anti-H	Ac	Bc	Oc	Phenotype	Genotype	GenBank ID
1	0	mf	2+	0	4+	4+	0	0	Bw	ABO*Bw.new/ABO*O.01.01	MW390872
2	4+	1+	4+	4+	3+	0	0	0	ABw	ABO*A1.02/ABO*Bw.new	
3	4+	1+	4+	0	3+	0	±	0	ABw	ABO*A2.05/ABO*Bw.new	OR373101

*ID number 1 and 2 with ABO*Bw.new was ABO*B.01 with c.98 + 3A>G. ID number 3 with ABO*Bw.new was ABO*B.01 with c.155 + 1G>T. Ac, A cells; Bc, B cells; Oc, O cells; mf, mixed-field agglutination; 0, no agglutination.

### Analysis of the full CDS regions of the *ABO* gene

**Figure 1:**
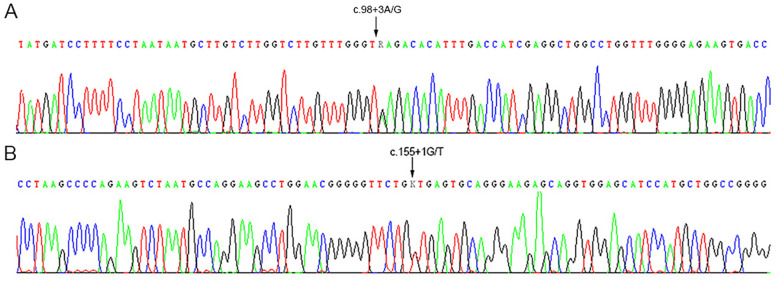
The heterozygous sequence of the splicing sites in the individuals with ABO subtypes. Arrows indicated the heterozygous variations in the *ABO* gene. **(A)** c.98 + 3A>G; **(B)** c.155 + 1G>T.

Specifically, after aligning the sequencing data to the ABO*A1.01 reference sequence, the following genotypes were identified. ID1: ABO*B.01 and ABO*O.01.01, with a heterozygous c.98 + 3A>G variant. ID2: ABO*A1.02 and ABO*B.01, with a heterozygous c.98 + 3A>G variant. ID3: ABO*A2.05 and ABO*B.01, with a heterozygous c.155 + 1G>T variant. These variants were located at the splicing donor sites (**Figure 1**). Based on the serological findings and the CDS sequence analysis, we propose that the splice-site variants are located on the ABO*B.01 allele. ABO genotypes of individuals based on the sequences of all exons are described in **Table 1**.

### Analysis of the *ABO* gene’s haplotype

**Figure 2:**
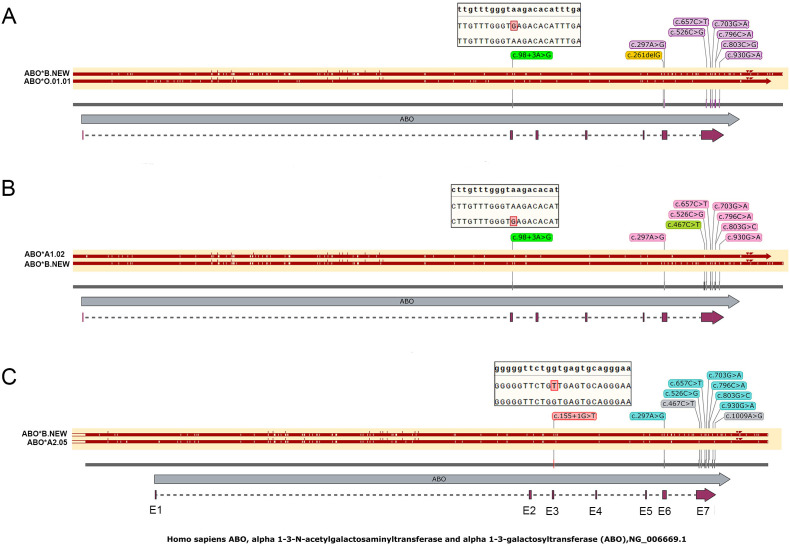
Haplotype analysis of ABO gene variants in three probands. **(A)** ID1 (Bw) with c.98 + 3A>G; **(B)** ID2 (ABw) with c.98 + 3A>G; **(C)** ID3 (ABw) with c.155 + 1G>T.

To formally determine whether the c.98 + 3A>G and c.155 + 1G>T variants are located on the B alleles, full-length *ABO* gene haplotype analysis was performed using an improved one-step ultra-LR-PCR, two haplotypes of the full-length *ABO* gene were obtained. The haplotype analysis confirmed that both splice-site variants reside on the B alleles. No other variants were detected in the coding regions, promoter, intron 1-specific regulatory element, or 3’ untranslated region (**Figure 2**). The c.98 + 3A>G variants were identified in ID1 and ID2 donors. The c.155 + 1G>T variant was identified in ID3 donor (**Table 2**). The sequences for the novel variants were submitted to the GenBank Database, and accession numbers were listed in **Table 1**.

**Table 2:** PacBio single-molecule sequencing results of ABO gene for the probands.

ID	Gene	Haplotype 1			Haplotype 2			Phenotype
		Phenotype	Allele	Nucleotide change	Phenotype	Allele	Nucleotide change	
1	*ABO*	O	ABO*O.01.01	c.261delG	Bw	ABO*Bw.new	c.98 + 3A>G;c.297A>G;c.526C>G;c.657C>T;c.703G>A;c.796C>A;c.803G>C;c.930G>A	Bw
2	*ABO*	A1	ABO*A1.02	c.467C>T	Bw	ABO*Bw.new	c.98 + 3A>G;c.297A>G;c.526C>G;c.657C>T;c.703G>A;c.796C>A;c.803G>C;c.930G>A	A1Bw
3	*ABO*	A2	ABO*A2.05	c.467C>T; c.1009A>G	Bw	ABO*Bw.new	c.155 + 1G>T;c.297A>G;c.526C>G;c.657C>T;c.703G>A;c.796C>A;c.803G>C;c.930G>A	A2Bw

### In silico prediction of splicing effects

Both variants were predicted to impact splicing, albeit with varying probabilities depending on the algorithm used (**Table 3**). Analysis with the SpliceAI predicted donor loss probabilities (Δ scores) of 0.37 and 0.13 for the c.98 + 3A>G and c.155 + 1G>T variants. In agreement, all four algorithms integrated in Alamut Visual Plus (SpliceSiteFinder-like, MaxEntScan, NNSPLICE, and GeneSplicer) consistently predicted a reduction in splice site strength, particularly for the canonical c.155 + 1G>T variant, which was scored as completely abolished by most tools.

**Table 3:** The splice probability of the variant types in silico analysis using different tools.

Variants	ISBT allele name	GenBank ID number	gnomAD v2.1.1 ID number#	dbSNP ID number	Frequency	Nearest type	SSF (0–100)			MaxEnte(0–12)			NNS(0–1)			GS(0–24)			SpliceAI
							Score		Change %	Score		Change %	Score		Change %	Score		Change %	ΔScore
							WT	MUT		WT	MUT		WT	MUT		WT	MUT		Donor loss
c.98 + 3A>G	Novel	MW390872	g.136137499T>C	Not referenced	NA	5’	81.6	77.2	5.40%	8.9	6	32.58%	1	0.5	50%	3.1	0.9	70.97%	0.37
c.155 + 1G>T	Novel	OR373101	g.136136720C>A	rs1554758180	NA	5’	87.1	0	100%	10.1	0	100%	1	0	100%	9	0	100%	0.13

*SSF, SpliceSiteFinder-like; MaxEnte, MaxEntScan; NNS, NNSPLICE; GS, GeneSplicer; WT, wild type; MUT, mutation.

NA not applicable. #The position in gnomAD v2.1.1 is referenced from NC_000009.11 in gnomAD v2.1.1. Value in parentheses of the tools is score range. Nucleotide position 1 in the variants is identical to the first nucleotide of the coding sequence.

### Splice-site variants result in exon skipping in *in vitro* assays

**Figure 3:**
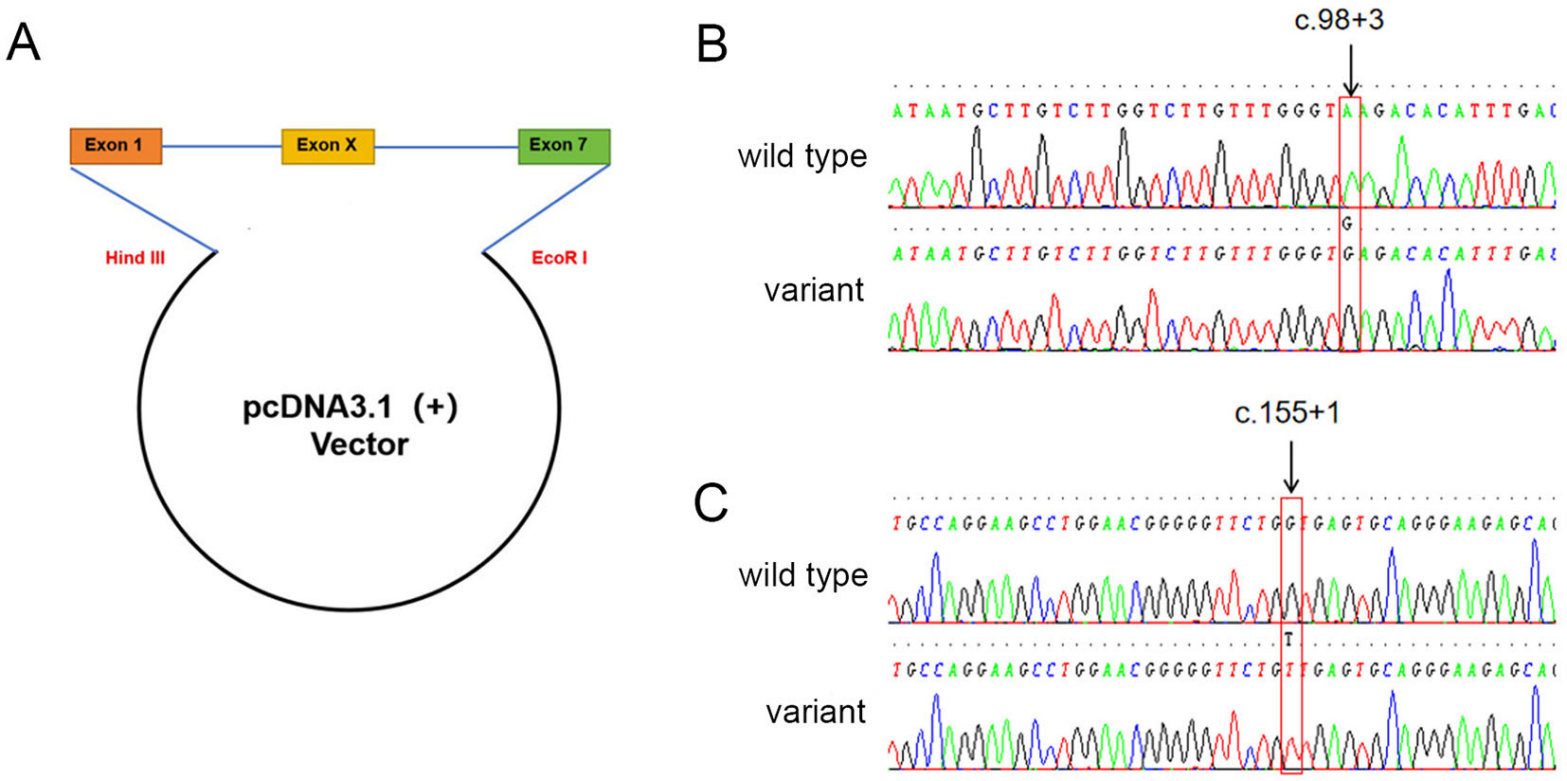
Minigene reporter plasmid sequencing results. **(A)** Schematic diagram of the minigene construct; **(B)** Sanger sequencing chromatograms of the wild type (c.98 + 3A) and variant (c.98 + 3G) plasmids; **(C)** Sanger sequencing chromatograms of the wild type (c.155 + 1G) and variant (c.155 + 1T) plasmids.

**Figure 4:**
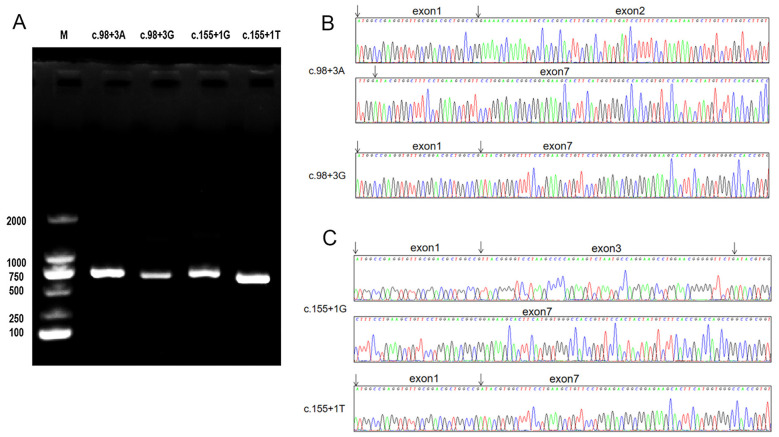
Minigene product sequencing. The image shown represents three independent experiments. **(A)** Electrophoresis diagram after rubber tapping and recovery following RT-PCR. **(B)** Sanger sequencing confirmation of exon 2 skipping induced by the c.98 + 3A>G variant; **(C)** Sanger sequencing confirmation of exon 3 skipping induced by the c.155 + 1G>T variant.

Four recombination plasmids (c.98 + 3A>G, c.155 + 1G>T, wild-type control c.98 + 3A and c.155 + 1G) were constructed, and the sequences of them were the same as designed (**Figure 3**). They were transfected into K562 cells, and specific ABO cDNA was analyzed. Agarose gel electrophoresis showed shortened PCR product from the cells transected with the expression plasmids containing c.98 + 3A>G or c.155 + 1G>T compared to wild-type controls (**Figure 4A**). The sequencing results revealed that the c.98 + 3A>G variant led to exon 2 deletion (**Figure 4B**), while the c.155 + 1G>T variant led to exon 3 deletion (**Figure 4C**). The intensity analysis on the agarose gel bands in **Figure 4A** was performed using the quantification tool in Image Lab 6.1 software. The adjusted volume results are as follows: c.98 + 3G band intensity was approximately 60% lower than c.98 + 3A, whereas the c.155 + 1T band intensity was approximately 60% higher than that of c.155 + 1G.

### B antigen expression was significantly weakened in HeLa cells transfected with ABO*B.01 cDNA without exons 2 or 3 *in vitro*

**Figure 5:**
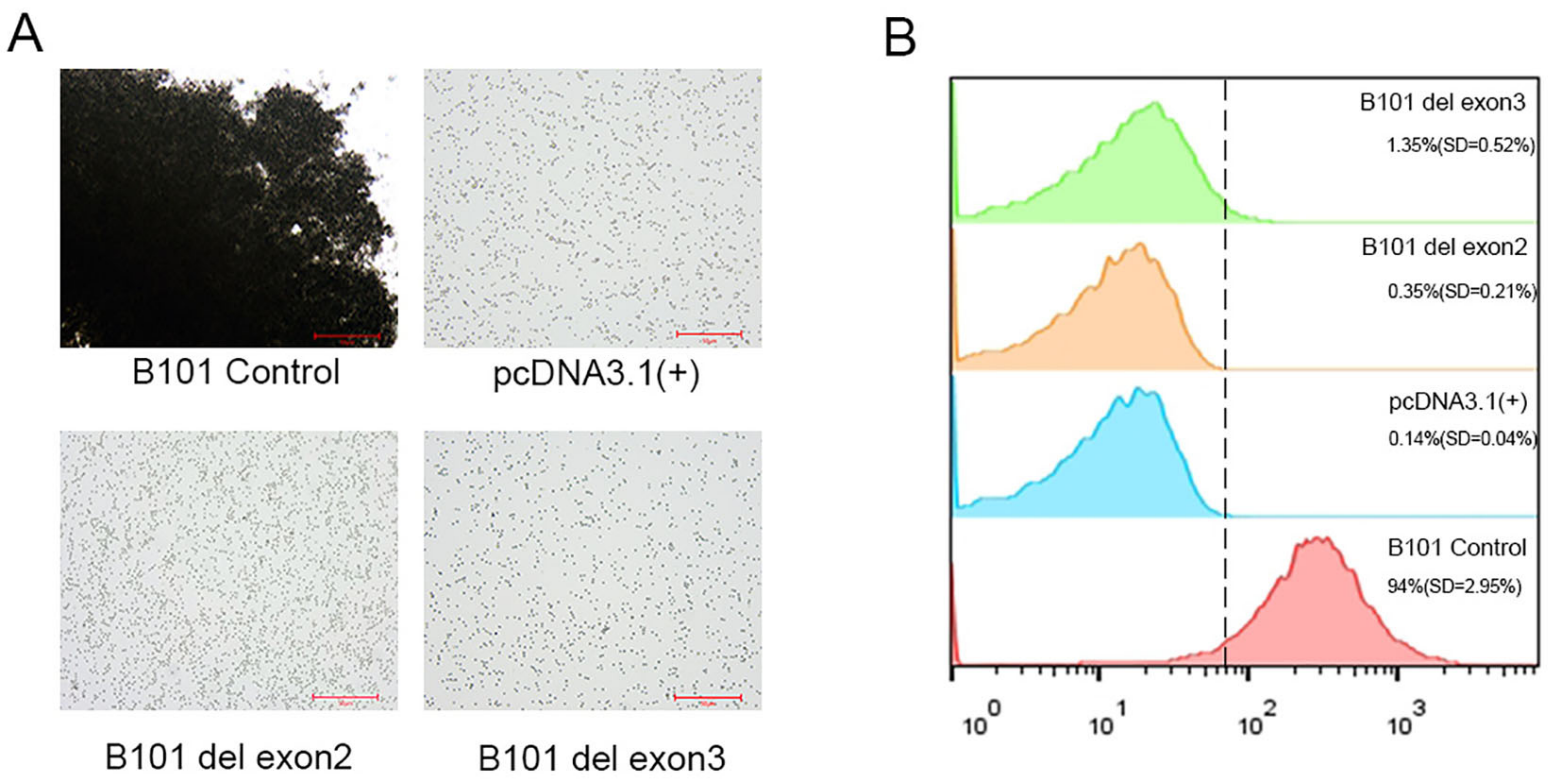
Expression of B antigen on the surface of cells transfected with different ABO cDNA variants representing exon skipping effects.The image shown represents three independent experiments. **(A)** Serology test(scale bar=50 µm); **(B)** flow cytometry.

The cells exhibited no agglutination with anti-B antibody using the serological method. Flow cytometry revealed that B antigen expression was virtually undetectable in cells transfected with constructs lacking exons 2 or 3 (**Figure 5**), which is lower than that of the positive control (*p* < 0.0001). In contrast, cells transfected with the ABO*B.01 wild-type positive control exhibited 4+ strength agglutination with anti-B antibody by the serological method, and strong B antigen expression by flow cytometry.

### No enzyme activity was observed in the supernatants of HeLa cells transfected with ABO*B.01 cDNA without exons 2 or 3 *in vitro*

The supernatant of ABO*B.01 cDNA without exon 2 or 3 demonstrated no agglutination after 4 or 24 h of incubation. While the supernatant of ABO*B.01 constitutive cDNA displayed agglutination at either 4 or 24 h; the titre of GTB activity in culture supernatants is depicted in **Supplementary Table 4**, **Supplementary Figure 1**.

### Comparable mRNA expression levels across constructs

qRT-PCR analysis revealed no significant difference in mRNA levels among ABO*B.01 cDNA and ABO*B.01 cDNA without exons 2 or 3 (one-way ANOVA, *p* = 0.8044) (**Supplementary Table 3**). This indicates that transcriptional efficiency or mRNA stability was not compromised by the deletions.

## Discussion

In addition to common A, B, O, and AB phenotypes, a wide variety of ABO subtypes exist within the populations, making the ABO blood group system more complex and diverse (6, 7). These ABO subtypes are prone to misidentification, which can lead to transfusion reactions (4, 5). Systematic investigation of these ABO subtypes and a comprehensive study of the molecular mechanisms of differential antigen expression can provide a foundation for regulating blood group antigen expression and contribute to enhanced transfusion safety.

Although many splice-site variants were identified in ABO subtype previously, functional research was performed on few (20, 29, 30). In this study, c.98 + 3A>G and c.155 + 1G>T variants at the *ABO* gene intron splicing sites were identified in three B-subtype probands. On the ABO*B.01 haplotype, no other variants were detected in the protein-coding region, promoter, intron 1-specific regulatory elements, or the 3’ untranslated region (3’ UTR) through full-length sequencing.

Theoretically, variants at intron splice sites or adjacent positions can generate novel RNA splice variants, leading for example to the loss of neighboring exons.Therefore, such predictions require *in vitro* validation. RNA splicing is a crucial biological process in eukaryotic gene expression, where introns are removed, and exons are concatenated to produce functional, protein-coding mRNAs (23, 24, 31). This process is a key mechanism for generating protein diversity in higher eukaryotes. Splicing site variants inevitably alter splicing patterns, leading to exon skipping or activation of nearby cryptic splice sites. Consequently, mature RNA may retain intronic sequences or lose exonic regions and produce partially functional or nonfunctional proteins during translation. Hence, normal gene expression is disrupted (32, 33).

To determine the impact of c.98 + 3A>G and c.155 + 1G>T variants on transcription, several experiments were performed. Bioinformatic analysis of these variants using multiple algorithms (**Table 3**) yielded quantitatively variable score changes. Traditional algorithms are highly tuned to the core splice consensus motif (GT-AG), making them exquisitely sensitive to variations at the invariant +1 position, often predicting entirely disruptive effects. In contrast, SpliceAI is trained on a broader genomic context and may better capture nuanced effects or the potential for “leaky” splicing, especially for non-canonical positions like +3. Particularly, in the case of c.98 + 3A>G variant, predictions ranged from a slight decrease to complete loss of splice site strength. This variability reflects the distinct underlying algorithms and training datasets of each tool. For instance, MaxEntScan is highly sensitive to core splice site consensus sequences, while NNSPLICE incorporates a broader context. Despite the numerical disparity, the concordant direction of all predictions toward a reduction in splice site strength provides a strong rationale for experimental investigation.

The effects of variants on *ABO* gene splicing were validated by the minigene technique *in vitro*. The minigene reporter plasmid was constructed by ligating ABO exons 1, X, and 7, along with their flanking intronic sequences, into the pcDNA3.1(+) backbone, encompassing the predicted splice sites. Exon X corresponds to the exon adjacent to the variant site. The minigene construct was designed to contain only the essential elements for splicing analysis: exon 1 (containing the translation start codon), the exon of interest with its flanking intronic sequences, and exon 7 (containing the stop codon). This minimal design was necessitated by the principles for splicing assays and is standard practice for assessing splice site function.Wild-type and mutant minigene reporter plasmids for each splice site variant were successfully generated and electroporated into K562 cells. ABO-specific PCR amplification was performed, and the products were analyzed by agarose gel electrophoresis followed by sequencing to assess the impact of intronic variants on splicing patterns *in vitro*. The results suggested that c.98 + 3A>G and c.155 + 1G>T variants affect the transcript. The intensity of agarose gel bands in the minigene assay was used for semi-quantitative analysis in this study. However, cDNA templates were not quantitatively normalized prior to PCR, and amplification efficiencies may differ between the wild-type (exons 1-2–7 or 1-3-7) and mutant (exons 1-7) amplicons. Therefore, semi-quantitative data may not allow to assess splicing efficiency.

To evaluate the function of glycosyltransferase B, an ABO expression system was established *in vitro.* Using serological testing, flow cytometry, and enzymatic activity assays, B antigen expression on transfected cell surfaces and the enzymatic activity of proteins secreted into culture supernatants were analyzed. The results revealed that the splicing variants that skipped exons 2 or 3 had no B antigen expression. In this study, *in vitro* analysis demonstrated that exon 2 deletion disrupts the reading frame and leads to premature termination. This underscores the critical role of exon 2 and suggests that residual B antigen in patients likely arises from incomplete splicing defects. Exon 3 deletion leads to the loss of 19 amino acids, which is predicted to alter the spatial conformation of glycosyltransferase. Comparable mRNA expression across all constructs (**Supplementary Table 3**) rules out transcriptional downregulation as the cause of the observed loss of B antigen and GTB activity. Consequently, the severe functional impairment arises from post-transcriptional mechanisms. For exon 2 deletion, the frameshift leads to a premature stop codon, predicting a truncated protein product that is likely to lack enzymatic activity. For exon 3 deletion, the loss of 19 amino acids within a critical structural domain is predicted to cause misfolding and loss of enzymatic activity (29). This is consistent with the almost complete absence of B antigen on the cell surface (**Figure 5**) and the null GTB activity in supernatants (**Supplementary Table 4**), which together establish these variants as severe loss-of-function alleles at the protein level.

The phenotypic variability observed between ID1 (mixed-field) and ID2 (1+), despite sharing the same c.98 + 3A>G variant, can be attributed to differences in splicing efficiency and genetic backgrounds. The c.98 + 3A>G variant likely reduces but does not completely abolish the correct splicing *in vivo*. Consequently, very little full-length mRNA may be present in the specimens. The mixed-field agglutination in ID1 suggests that a proportion of erythrocytes may produce a normal amount of B antigen, whereas another proportion produces very little or none-a pattern consistent with stochastic “leaky” splicing resulting in two erythrocyte populations. In contrast, the uniform weak (1+) reaction in ID2 may reflect a homogeneous splicing efficiency with a higher proportion of full-length mRNA than ID1. Furthermore, ID2’s ABw background has the co-dominant A allele. The efficient A transferase may compete for the common H antigen precursor with the weakened B enzyme, potentially further modulating the final B antigen and H antigen density detected serologically.

Our *in vitro* expression analysis confirmed the loss of function with complete exon deletion, either via premature termination (exon 2) or critical structural disruption (exon 3). However, in patients, low-level “leaky” splicing may produce a small amount of full-length functional transcript, accounting for the trace B antigen observed. This interpretation is supported by previous reports of leaky splice variants that generate functional transcripts (34). In addition, alternative translation initiation from downstream exons was found in some genes (35), including in weak ABO phenotypes (36) and represents a theoretical possibility in our specimens (**Supplementary Figure 2**); however, no evidence for such products was detected in our experimental system, and further investigation is warranted.

The occurrence of these variants highlights a critical need for enhanced diagnostic protocols. We recommend that serological findings of weak or mixed-field agglutination with anti-B antibody reagents should prompt molecular investigation, especially in populations where these variants may be present. Only standard sequencing of ABO exons is insufficient; targeted sequencing of intronic regions flanking exons 2-6, complemented by *in silico* splicing prediction, is essential. For the probands identified with such variants, family studies can clarify inheritance and aid in genetic counseling.

These findings have direct implications for transfusion safety. Misclassification of a weak B subtype as group O could lead to the selection of an incompatible unit if the recipient has a corresponding anti-B antibody. Although a high titer of anti-B antibody in such individuals is rare, the risk of misclassification is not zero. In cases of discrepant or weak serology, establishing algorithms for molecular typing is advisable for the blood centers serving ethnically diverse populations. It is also particularly crucial for donor units intended for neonatal exchange transfusion or immunocompromised recipients.

Beyond transfusion medicine, precise characterization of splice variants has broader implications. (1) These alleles can serve as natural models for studying pre-mRNA splicing regulation in a clinically relevant context. (2)With the advances in genomic medicine, understanding the full spectrum of ABO variation is critical for accurate interpretation of genome sequencing data in the context of polygenic risk factor or pharmacogenomics, where ABO status is a collaborative factor (for example, in von Willebrand factor levels or cardiovascular risk). (3) Engineered nucleases or splicing modulators designed for other diseases must account for such splice sites to avoid off-target effects on genes like *ABO*.

This study has certain limitations that should be acknowledged. Although the number of probands is small (two with c.98 + 3A>G and one with c.155 + 1G>T), this is not unexpected for rare variants and does not diminish the functional causality established here. The rarity of these variants is supported by the gnomAD database, where both alleles are absent or have no reported frequency (not referenced/NA). It suggests that these variants may only exist in individuals with rare ABO subtypes. A more relevant limitation is that the splicing products in the minigene assay were analyzed by Sanger sequencing, which may not detect rare transcripts present at very low abundance. cDNA-sequencing with NGS would offer greater sensitivity and enable precise quantification of the relative proportions of correctly spliced and aberrant transcripts, particularly for assessing low-level “leaky” splicing.

This study provides the conclusive functional evidence that these specific variants (c.98 + 3A>G and c.155 + 1G>T) are pathogenic via exon skipping. The combined evidence from haplotype assignment, *in silico* prediction, minigene assays, and stable cell line expression forms a robust and consistent chain of evidence that does not require large sample sizes. The assays robustly establish the causative link between genotype and splicing defect although splicing efficiency *in-vivo* may be modulated by additional trans-acting factors in the full genomic context.

Future studies are warranted to ascertain the true population frequency and to discover additional carriers by screening larger blood donor cohorts or aggregating data from regional blood centers.

## Conclusions

In summary, two intronic splice-site variants, c.98 + 3A>G and c.155 + 1G>T, were found in three Chinese blood donors. These variants induce aberrant mRNA splicing, resulting in exons 2 or 3 skipping and producing a nonfunctional glycosyltransferase.

## Data Availability

The datasets presented in this study can be found in online repositories. The names of the repository/repositories and accession number(s) can be found in the article/**Supplementary Material**.
