# Supplementary material for: c.98 + 3A>G and c.155 + 1G>T splice-site variants in the ABO*B.01 allele lead to weak antigen expression in the Chinese individuals
Source: Front Immunol. 2026 Apr 22;17:1739842. doi: 10.3389/fimmu.2026.1739842 (PMC13143704; doi:10.3389/fimmu.2026.1739842)
Supplement: Supplementary file 1 [file DataSheet1.docx]

Supplementary Materials

Supplementary Methods

# RT-PCR analysis for ABO cDNA in the K562 cells with minigene reporter plasmid

Total RNAs were isolated from transfected cells using the QIAamp® RNA Blood Mini Kit (Qiagen Company, Hilden,Germany) according to the manufacturer,s instruction. RNA were reverse transcribed into cDNA using the PrimeScritTM Reagent Kit (RR037A,Takara,Dalian,China). Briefly, first-strand cDNA synthesis was carried out in a 20 μL reaction mixture containing 4 μL of 5X PrimeScript Buffer, 1 μL of PrimeScript RT Enzyme Mix I, 1 μL of Oligo dT Primer, 1 μL of Random 6 mers, and 2 μg of total RNA. Nuclease-free water was added to a final volume of 20 μL. The reaction was incubated at 37°C for 15 minutes, followed by enzyme inactivation at 85°C for 5 seconds. Subsequently, 2 μL of RNase H was added to the product, which was then further incubated at 37°C for 20 minutes.

The cDNA was then amplified using the primers and 2x TransStart® FastPfu Fly PCR SuperMix kit (AS231, TransGen Biotech,Beijing,China), the sequences of the forward and reverse primer were 5^,^ ATGGCCGAGGTGTTGCGGAC 3^,^ and 5^,^ TAATCCACCTCGCTGAGGAA 3^,^ which were located in the exon 1 and exon 7 respectively.A 50 μL reaction mixture was prepared containing 25 μL of 2x SuperMix, 1 μL of each forward and reverse primer (10 μM), 2 μL of cDNA template, and 21 μL of nuclease-free water. PCR cycling conditions were carried out according to the manufacturer's recommendations for amplification of the target fragment.PCR amplification was performed under the following thermal cycling conditions: an initial denaturation at 95°C for 2 min; followed by 35 cycles of denaturation at 95°C for 20 s, annealing at 60°C for 20 s, and extension at 72°C for 20 s; with a final extension step at 72°C for 5 min. Amplions were separated by electrophoresis through an agarose gel, and the DNA size marker is used as control (Takara,Dalian,China). The gel of each ABO cDNA band in the K562 cells was cut and purified using QIAquick® Gel Extraction Kit (Qiagen Company, Hilden,Germany) , then sequenced and analyzed as the description in the "Construct minigene reporter plasmid" section. All experiments were performed with at least three independent biological replicates.

# Analysis B antigen by flow cytometry

Stable HeLa cells that have been screened by G418 for stable expression were harvested, washed twice with phosphate-buffered saline (PBS), and resuspended in PBS at a density of approximately 1×10⁶ cells per 100 µL. For surface B antigen detection, cells were stained with a phycoerythrin (PE)-conjugated monoclonal mouse anti-human B antigen antibody ( Vendor: ARP; Catalog #: 08-9431-4) at a dilution of 1:500 in the dark for 30 minutes.

Flow cytometry was performed using a BD FACSVerse Flow cytometer. For each sample, a minimum of 10,000 events within the target cell population were collected. Data were acquired using BD FACSuite software. Data were analyzed using FlowJo v10.8. The analysis was performed using a two-step gating strategy : Gate 1 (Target Cell Population): Cells were first plotted on a Forward Scatter (FSC-A) versus Side Scatter (SSC-A) dot plot. The homogeneous population of intact, healthy cells (characterized by high FSC and intermediate SSC) was gated (P1). This gate effectively excludes small debris (low FSC and SSC) and large aggregates. Gate 2 (B Antigen-Positive Population): The cells from P1 were then analyzed for PE fluorescence intensity on a histogram. The negative fluorescence threshold was rigorously set using the pcDNA3.1(+) sample so that >99% of its events fell within the negative region. For experimental samples, the percentage of B antigen-positive cells was calculated as the proportion of events in P1 that displayed fluorescence intensity above this established threshold. All experiments were performed with at least three independent biological replicates.

# B glycosyltransferase activity assay of the transfected cells

**3.1. Preparation of Reaction Components**

1× Reaction Buffer: The assay buffer (50 mL final volume) was freshly prepared containing 50 mM imidazole, 150 mM NaCl, 25 mM MnCl₂, and 0.5% (w/v) bovine serum albumin (BSA). The pH was adjusted to 6.5. The buffer was filter-sterilized (0.22 µm) prior to use.

Substrate Solution: The nucleotide sugar donor, UDP-galactose (UDP-Gal, Sigma U4500), was dissolved in nuclease-free water to a stock concentration of 5 mg/mL. Aliquots of 60.9 µL were prepared and stored at -20°C. For each assay, an aliquot was thawed and diluted with 1× Reaction Buffer to a final working concentration of 0.5 mM UDP-Gal.

**3.2. Enzymatic Reaction**

Washed, packed group O RBCs were used as the acceptor substrate. For each test sample, a 160 µL reaction mixture was set up in a microcentrifuge tube containing: 10 µL of packed O RBCs, 100 µL of the 0.5 mM UDP-Gal substrate working solution, and 50 µL of cell culture supernatant collected from transfected HeLa cell clones. The mixture was gently vortexed and incubated at 37°C. Parallel reactions were set up for two time points: 4 hours and 24 hours. A negative control reaction, substituting the test supernatant with supernatant from empty vector-transfected cells, was included in each experiment.

**3.3. Detection and Titer Determination**

Following incubation, the RBCs were washed three times with 0.9% normal saline to stop the reaction and remove excess reagents. The washed RBCs were resuspended in normal saline to a 0.8% (v/v) concentration for serological testing. A 50 µL aliquot of the resuspended RBCs was mixed with 25 µL of monoclonal anti-B typing reagent in the reaction chamber of a standard microcolumn gel card. The card was incubated at room temperature for 15 minutes and then centrifuged in a dedicated centrifuge (DiaMed ID-Centrifuge) for 10 minutes according to the manufacturer's instructions. Agglutination results were read visually. The appearance of a positive agglutination reaction indicated the presence of newly synthesized B antigen, confirming GTB activity in the supernatant. To determine the enzymatic titer, positive samples were subjected to endpoint titration.

All experiments were performed with at least three independent biological replicates.

**4 Quantitative Real-Time PCR (qRT-PCR)**

Total RNAs were isolated from transfected cells using the QIAamp® RNA Blood Mini Kit (Qiagen Company, Hilden,Germany) according to the manufacturer,s instruction. RNA were reverse transcribed into cDNA using the PrimeScritTM Reagent Kit (RR037A,Takara,Dalian,China). Briefly, first-strand cDNA synthesis was carried out in a 20 μL reaction mixture containing 4 μL of 5X PrimeScript Buffer, 1 μL of PrimeScript RT Enzyme Mix I, 1 μL of Oligo dT Primer, 1 μL of Random 6 mers, and 2 μg of total RNA. Nuclease-free water was added to a final volume of 20 μL. The reaction was incubated at 37°C for 15 minutes, followed by enzyme inactivation at 85°C for 5 seconds. Subsequently, 2 μL of RNase H was added to the product, which was then further incubated at 37°C for 20 minutes.

mRNA expression was quantified by TaqMan probe-based qRT-PCR using a duplex assay that co-amplified the target gene and the endogenous control (GAPDH) in the same reaction. The target transcript (261-G) was detected using a FAM-labeled probe, and the GAPDH transcript was detected using a VIC-labeled probe. The 20 µL reaction mixture consisted of:10 µL of 2× SGExcel GoldStar TaqMan Mixture(Sangon Biotech,Shanghai,China);1.2 µL of each forward and reverse primer (10 µM) for the 261-G target,the sequences of the forward and reverse primer were 5^，^GCAGAAGCTGAGTGGAGTTTC 3^，^and 5^，^GACAATGGGAGCCAGCC 3^，^; 0.1 µL of each forward and reverse primer (10 µM) for the GAPDH target; 0.2 µL of the FAM-labeled probe (10 µM) for 261-G,the sequences were 5^，^FAM-AAGAATGTCCTCGTGGTGACCC 3^，^-BHQ1; 0.1 µL of the VIC-labeled probe (10 µM) for GAPDH; 5.1 µL of nuclease-free water, and 2.0 µL of cDNA template. The thermal cycling protocol was performed as follows: an initial hold at 95°Cfor 3 minutes, followed by 40 cycles of denaturation at 95°C for 20 seconds and combined annealing/extension at 60°C for 30 seconds. Fluorescence data were collected at the end of each 60°C step.

Supplementary Results

**Supplemental Table S1**  Discontinuous sequences of the *ABO* gene were cloned into the pcDNA3.1(+) vector.

| Vector | Sequences |
| --- | --- |
| 98+3A | CTCTGTCCCCTCCCGTGTTCGGCCTCGGGAAGTCGGGGCGGCGGGCGGCGCGGGCCGGGAGGGGGCGCCTCGGGCTCACCCCGCCCCAGGGCCGCCGGGCGGAAGGCGGAGGCCGAGACCAGACGCGGAGCCATGGCCGAGGTGTTGCGGACGCTGGCCGGTGAGTGCAGGCCTCGGCCCCGGGTGCCCGCGAGGGAGCCGCTACCGCAGGGAATGCGGGGTGCACCCGACAGCCGGGCCGGGGTGGGGGCGCTCAGGGCTGCGAGGCTTCGGGCCGGCCGCCGCCCCAGCCTCCGAGACCCTGCGTCCTGGGGAGCCGGCGGGCAGGTGGGCTCGGCCGCGCTGTGGGTGCCTGGGACCCGCAGGGAGGATGGGCGCGGTGGCGCGGCCTGGCGGGGGGCTCGTCTCCGGGGTCCCCGGGTCCTGGTGAGAGCGGGGTCCCTCGACGCCGTGGCGGTCTCCAGCCTCTCCTCGACCATCTTGGCAGATGAAGGCCCGTCGCAGGGTGTGATGCCTGAATTACAAGGCGGGACAGGTAAAGTGGGGCAGGTGAGAGAAGGAGGGTGAGTGATGTGATTTTTCTACTCCTGTTTTCCAGGAAAACCAAAATGCCACGCACTTCGACCTATGATCCTTTTCCTAATAATGCTTGTCTTGGTCTTGTTTGGGTAAGACACATTTGACCATCGAGGCTGGCCTGGTTTGGGGAGAAGTGACCACAGCAGCCAATCAGACCCATGGGGCCTCCCTGAGCTCCCCAAGTATCACAGTTATCAGGGTCCTAAGGACAGTTATTGCCTGCGTCCAGCTCTGGCGGAGGGTGTGCTTACTTGCTCCCTTATTTTAGCCTCACCTGGGCAACAGGCTCATCTCACTCCCATTTAAAATTTTCCTAAGTGTGGAGTCTGGGGCTGGGAGAGCAAGCCCCTTGCCCACAATTGCGTGGCTGGGGGTGGGGAAGGCAATTCTGGGTCCCAAGCTGTTAGTCGCTTCCAGACACAGAAGGTCCCAGAACCAAGAGTGAAGTCACCTGTCACCTCTACTGGGGCATCTCTGGACACGGTCTGGAAACACTCCCTGACGTGGCCTCAGGGACTGCACTGACCAAGGCACTGGTGGCGGGGGGTGAGGGAGCTGGGGCTCTGGAGCTCCAGCAGGTGCCCATACGTGAGCAATATCCCAGGGACCACCCTCCTGCCCACCTCCCGGTGTGGGACGTGGCGAGGCGCCTGAGCTTTGCTGAGAACTTGCCCTACCTGCCTCGAGGCCTTGCAGCTTCACCGGGAACTCTTGTGCTCACGCTGCTGGCCGCACCATGCACTTTTTGGAGGAAGGGACCAACAGGCAGTCTTCGTTCTGTGTCCTGAGTCTTGGCTCTGCTGCTCTAAGCCTTCCAATGGCCGCTGGCGGGCGGGTGCAGGACGGGCCTCCTGCAGCCCAGGGGTGCACGGCCGGCGGCTCCCCCAGCCCCCGTCCGCCTGCCTTGCAGATACGTGGCTTTCCTGAAGCTGTTCCTGGAGACGGCGGAGAAGCACTTCATGGTGGGCCACCGTGTCCACTACTATGTCTTCACCGACCAGCCGGCCGCGGTGCCCCGCGTGACGCTGGGGACCGGTCGGCAGCTGTCAGTGCTGGAGGTGGGCGCCTACAAGCGCTGGCAGGACGTGTCCATGCGCCGCATGGAGATGATCAGTGACTTCTGCGAGCGGCGCTTCCTCAGCGAGGTGGATTACCTGGTGTGCGTGGACGTGGACATGGAGTTCCGCGACCATGTGGGCGTGGAGATCCTGACTCCGCTGTTCGGCACCCTGCACCCCAGCTTCTACGGAAGCAGCCGGGAGGCCTTCACCTACGAGCGCCGGCCCCAGTCCCAGGCCTACATCCCCAAGGACGAGGGCGATTTCTACTACATGGGGGCGTTCTTCGGGGGGTCGGTGCAAGAGGTGCAGCGGCTCACCAGGGCCTGCCACCAGGCCATGATGGTCGACCAGGCCAACGGCATCGAGGCCGTGTGGCACGACGAGAGCCACCTGAACAAGTACCTACTGCGCCACAAACCCACCAAGGTGCTCTCCCCCGAGTACTTGTGGGACCAGCAGCTGCTGGGCTGGCCCGCCGTCCTGAGGAAGCTGAGGTTCACTGCGGTGCCCAAGAACCACCAGGCGGTCCGGAACCCGTGAGCGGCTGCCAGGGGCTCTGGGAGGGCTGCCGGCAGCCCCGTCCCCCTCCCGCCCTTGGTTTTAGCAGAACGGGTAAACTCTGTTTCCTTTGTCCGTCCTGTTGTGAGTAACTGAAGCCTAGGCCCC |
| 98+3G | CTCTGTCCCCTCCCGTGTTCGGCCTCGGGAAGTCGGGGCGGCGGGCGGCGCGGGCCGGGAGGGGGCGCCTCGGGCTCACCCCGCCCCAGGGCCGCCGGGCGGAAGGCGGAGGCCGAGACCAGACGCGGAGCCATGGCCGAGGTGTTGCGGACGCTGGCCGGTGAGTGCAGGCCTCGGCCCCGGGTGCCCGCGAGGGAGCCGCTACCGCAGGGAATGCGGGGTGCACCCGACAGCCGGGCCGGGGTGGGGGCGCTCAGGGCTGCGAGGCTTCGGGCCGGCCGCCGCCCCAGCCTCCGAGACCCTGCGTCCTGGGGAGCCGGCGGGCAGGTGGGCTCGGCCGCGCTGTGGGTGCCTGGGACCCGCAGGGAGGATGGGCGCGGTGGCGCGGCCTGGCGGGGGGCTCGTCTCCGGGGTCCCCGGGTCCTGGTGAGAGCGGGGTCCCTCGACGCCGTGGCGGTCTCCAGCCTCTCCTCGACCATCTTGGCAGATGAAGGCCCGTCGCAGGGTGTGATGCCTGAATTACAAGGCGGGACAGGTAAAGTGGGGCAGGTGAGAGAAGGAGGGTGAGTGATGTGATTTTTCTACTCCTGTTTTCCAGGAAAACCAAAATGCCACGCACTTCGACCTATGATCCTTTTCCTAATAATGCTTGTCTTGGTCTTGTTTGGGTGAGACACATTTGACCATCGAGGCTGGCCTGGTTTGGGGAGAAGTGACCACAGCAGCCAATCAGACCCATGGGGCCTCCCTGAGCTCCCCAAGTATCACAGTTATCAGGGTCCTAAGGACAGTTATTGCCTGCGTCCAGCTCTGGCGGAGGGTGTGCTTACTTGCTCCCTTATTTTAGCCTCACCTGGGCAACAGGCTCATCTCACTCCCATTTAAAATTTTCCTAAGTGTGGAGTCTGGGGCTGGGAGAGCAAGCCCCTTGCCCACAATTGCGTGGCTGGGGGTGGGGAAGGCAATTCTGGGTCCCAAGCTGTTAGTCGCTTCCAGACACAGAAGGTCCCAGAACCAAGAGTGAAGTCACCTGTCACCTCTACTGGGGCATCTCTGGACACGGTCTGGAAACACTCCCTGACGTGGCCTCAGGGACTGCACTGACCAAGGCACTGGTGGCGGGGGGTGAGGGAGCTGGGGCTCTGGAGCTCCAGCAGGTGCCCATACGTGAGCAATATCCCAGGGACCACCCTCCTGCCCACCTCCCGGTGTGGGACGTGGCGAGGCGCCTGAGCTTTGCTGAGAACTTGCCCTACCTGCCTCGAGGCCTTGCAGCTTCACCGGGAACTCTTGTGCTCACGCTGCTGGCCGCACCATGCACTTTTTGGAGGAAGGGACCAACAGGCAGTCTTCGTTCTGTGTCCTGAGTCTTGGCTCTGCTGCTCTAAGCCTTCCAATGGCCGCTGGCGGGCGGGTGCAGGACGGGCCTCCTGCAGCCCAGGGGTGCACGGCCGGCGGCTCCCCCAGCCCCCGTCCGCCTGCCTTGCAGATACGTGGCTTTCCTGAAGCTGTTCCTGGAGACGGCGGAGAAGCACTTCATGGTGGGCCACCGTGTCCACTACTATGTCTTCACCGACCAGCCGGCCGCGGTGCCCCGCGTGACGCTGGGGACCGGTCGGCAGCTGTCAGTGCTGGAGGTGGGCGCCTACAAGCGCTGGCAGGACGTGTCCATGCGCCGCATGGAGATGATCAGTGACTTCTGCGAGCGGCGCTTCCTCAGCGAGGTGGATTACCTGGTGTGCGTGGACGTGGACATGGAGTTCCGCGACCATGTGGGCGTGGAGATCCTGACTCCGCTGTTCGGCACCCTGCACCCCAGCTTCTACGGAAGCAGCCGGGAGGCCTTCACCTACGAGCGCCGGCCCCAGTCCCAGGCCTACATCCCCAAGGACGAGGGCGATTTCTACTACATGGGGGCGTTCTTCGGGGGGTCGGTGCAAGAGGTGCAGCGGCTCACCAGGGCCTGCCACCAGGCCATGATGGTCGACCAGGCCAACGGCATCGAGGCCGTGTGGCACGACGAGAGCCACCTGAACAAGTACCTACTGCGCCACAAACCCACCAAGGTGCTCTCCCCCGAGTACTTGTGGGACCAGCAGCTGCTGGGCTGGCCCGCCGTCCTGAGGAAGCTGAGGTTCACTGCGGTGCCCAAGAACCACCAGGCGGTCCGGAACCCGTGAGCGGCTGCCAGGGGCTCTGGGAGGGCTGCCGGCAGCCCCGTCCCCCTCCCGCCCTTGGTTTTAGCAGAACGGGTAAACTCTGTTTCCTTTGTCCGTCCTGTTGTGAGTAACTGAAGCCTAGGCCCC |
| 155+1G | CTCTGTCCCCTCCCGTGTTCGGCCTCGGGAAGTCGGGGCGGCGGGCGGCGCGGGCCGGGAGGGGGCGCCTCGGGCTCACCCCGCCCCAGGGCCGCCGGGCGGAAGGCGGAGGCCGAGACCAGACGCGGAGCCATGGCCGAGGTGTTGCGGACGCTGGCCGGTGAGTGCAGGCCTCGGCCCCGGGTGCCCGCGAGGGAGCCGCTACCGCAGGGAATGCGGGGTGCACCCGACAGCCGGGCCGGGGTGGGGGCGCTCAGGGCTGCGAGGCTTCGGGCCGGCCGCCGCCCCAGCCTCCGAGACCCTGCGTCCTGGGGAGCCGGCGGGCAGGTGGGCTCGGCCGCGCTGTGGGTGCCTGGGACCCGCAGGGAGGATGGGCGCGGTGGCGCGGCCTGGCGGGGGGCTCGTCTCCGGGGTCCCCGGGTCCTGGTGAGAGCGGGGTCCCTCGACGCCGTGGCGGTCTCCAGCCTCTCCTCGCCCCTCCACGCTCCCCGCCTTCCATGAGCTGCTATTTTCAGCACCTACCGCCTGTCCTGAGTCTTGGCACACTTCCTTTCTGCAGTTACGGGGTCCTAAGCCCCAGAAGTCTAATGCCAGGAAGCCTGGAACGGGGGTTCTGGTGAGTGCAGGGAAGAGCAGGTGGAGCATCCATGCTGGCCGGGGTGCTGGCTGTGGGCGGGGGTCCCACTCTGGGAACTCCCCCTCCCCTTCCTGGGCCCGCTCTCTATGCTCTGCCCAGTGTAGACATGCTCAACTCTGTGGCCTTGAAGGGTCACCTGGATACCTCTGGAGTCAGCCTTGACCTCCCTTCTGACCTCCAGTCCCCAACTCCAGGCTTACCCAGAGTTCCCATGCATGGTCTCTGCTCCCCCATCCCCACCCCTCCTCCACCAGCCATCCTCAACTCTCCCTTTGCTCTCACCCCTACACTGGGTTCCCAAATCCTGCCAGCCCTGCTCTTCAGCAGCTCGCGTGCCCACTGGCCTCCCTGCCTTCGACGTCCTGCTCCAGGGCTCCGGTGGGGCTGCCCTCATCTCTGTGACAGCCTCAGACTTGTCTCCCCATCCCCATCAGCCCTGTCCCCCTTCTTCTCACAGCAGCTGGAGTGATCTTTCCAGAACATAAGGTAGGAGGCTCTCCATGATCTGATCCTGCCTTCCTGACCTCCCCAGCCTCATCTCTCCCTCCTCTGCCCTCGCCCTCTGTGCTCCAGCCAACGTGGCCTGTCACTCGTCCACCTGCCATACTGTCCTGATCTGCTGCTCTAAGCCTTCCAATGGCCGCTGGCGGGCGGGTGCAGGACGGGCCTCCTGCAGCCCAGGGGTGCACGGCCGGCGGCTCCCCCAGCCCCCGTCCGCCTGCCTTGCAGATACGTGGCTTTCCTGAAGCTGTTCCTGGAGACGGCGGAGAAGCACTTCATGGTGGGCCACCGTGTCCACTACTATGTCTTCACCGACCAGCCGGCCGCGGTGCCCCGCGTGACGCTGGGGACCGGTCGGCAGCTGTCAGTGCTGGAGGTGGGCGCCTACAAGCGCTGGCAGGACGTGTCCATGCGCCGCATGGAGATGATCAGTGACTTCTGCGAGCGGCGCTTCCTCAGCGAGGTGGATTACCTGGTGTGCGTGGACGTGGACATGGAGTTCCGCGACCATGTGGGCGTGGAGATCCTGACTCCGCTGTTCGGCACCCTGCACCCCAGCTTCTACGGAAGCAGCCGGGAGGCCTTCACCTACGAGCGCCGGCCCCAGTCCCAGGCCTACATCCCCAAGGACGAGGGCGATTTCTACTACATGGGGGCGTTCTTCGGGGGGTCGGTGCAAGAGGTGCAGCGGCTCACCAGGGCCTGCCACCAGGCCATGATGGTCGACCAGGCCAACGGCATCGAGGCCGTGTGGCACGACGAGAGCCACCTGAACAAGTACCTACTGCGCCACAAACCCACCAAGGTGCTCTCCCCCGAGTACTTGTGGGACCAGCAGCTGCTGGGCTGGCCCGCCGTCCTGAGGAAGCTGAGGTTCACTGCGGTGCCCAAGAACCACCAGGCGGTCCGGAACCCGTGAGCGGCTGCCAGGGGCTCTGGGAGGGCTGCCGGCAGCCCCGTCCCCCTCCCGCCCTTGGTTTTAGCAGAACGGGTAAACTCTGTTTCCTTTGTCCGTCCTGTTGTGAGTAACTGAAGCCTAGGCCCC |
| 155+1T | CTCTGTCCCCTCCCGTGTTCGGCCTCGGGAAGTCGGGGCGGCGGGCGGCGCGGGCCGGGAGGGGGCGCCTCGGGCTCACCCCGCCCCAGGGCCGCCGGGCGGAAGGCGGAGGCCGAGACCAGACGCGGAGCCATGGCCGAGGTGTTGCGGACGCTGGCCGGTGAGTGCAGGCCTCGGCCCCGGGTGCCCGCGAGGGAGCCGCTACCGCAGGGAATGCGGGGTGCACCCGACAGCCGGGCCGGGGTGGGGGCGCTCAGGGCTGCGAGGCTTCGGGCCGGCCGCCGCCCCAGCCTCCGAGACCCTGCGTCCTGGGGAGCCGGCGGGCAGGTGGGCTCGGCCGCGCTGTGGGTGCCTGGGACCCGCAGGGAGGATGGGCGCGGTGGCGCGGCCTGGCGGGGGGCTCGTCTCCGGGGTCCCCGGGTCCTGGTGAGAGCGGGGTCCCTCGACGCCGTGGCGGTCTCCAGCCTCTCCTCGCCCCTCCACGCTCCCCGCCTTCCATGAGCTGCTATTTTCAGCACCTACCGCCTGTCCTGAGTCTTGGCACACTTCCTTTCTGCAGTTACGGGGTCCTAAGCCCCAGAAGTCTAATGCCAGGAAGCCTGGAACGGGGGTTCTGTTGAGTGCAGGGAAGAGCAGGTGGAGCATCCATGCTGGCCGGGGTGCTGGCTGTGGGCGGGGGTCCCACTCTGGGAACTCCCCCTCCCCTTCCTGGGCCCGCTCTCTATGCTCTGCCCAGTGTAGACATGCTCAACTCTGTGGCCTTGAAGGGTCACCTGGATACCTCTGGAGTCAGCCTTGACCTCCCTTCTGACCTCCAGTCCCCAACTCCAGGCTTACCCAGAGTTCCCATGCATGGTCTCTGCTCCCCCATCCCCACCCCTCCTCCACCAGCCATCCTCAACTCTCCCTTTGCTCTCACCCCTACACTGGGTTCCCAAATCCTGCCAGCCCTGCTCTTCAGCAGCTCGCGTGCCCACTGGCCTCCCTGCCTTCGACGTCCTGCTCCAGGGCTCCGGTGGGGCTGCCCTCATCTCTGTGACAGCCTCAGACTTGTCTCCCCATCCCCATCAGCCCTGTCCCCCTTCTTCTCACAGCAGCTGGAGTGATCTTTCCAGAACATAAGGTAGGAGGCTCTCCATGATCTGATCCTGCCTTCCTGACCTCCCCAGCCTCATCTCTCCCTCCTCTGCCCTCGCCCTCTGTGCTCCAGCCAACGTGGCCTGTCACTCGTCCACCTGCCATACTGTCCTGATCTGCTGCTCTAAGCCTTCCAATGGCCGCTGGCGGGCGGGTGCAGGACGGGCCTCCTGCAGCCCAGGGGTGCACGGCCGGCGGCTCCCCCAGCCCCCGTCCGCCTGCCTTGCAGATACGTGGCTTTCCTGAAGCTGTTCCTGGAGACGGCGGAGAAGCACTTCATGGTGGGCCACCGTGTCCACTACTATGTCTTCACCGACCAGCCGGCCGCGGTGCCCCGCGTGACGCTGGGGACCGGTCGGCAGCTGTCAGTGCTGGAGGTGGGCGCCTACAAGCGCTGGCAGGACGTGTCCATGCGCCGCATGGAGATGATCAGTGACTTCTGCGAGCGGCGCTTCCTCAGCGAGGTGGATTACCTGGTGTGCGTGGACGTGGACATGGAGTTCCGCGACCATGTGGGCGTGGAGATCCTGACTCCGCTGTTCGGCACCCTGCACCCCAGCTTCTACGGAAGCAGCCGGGAGGCCTTCACCTACGAGCGCCGGCCCCAGTCCCAGGCCTACATCCCCAAGGACGAGGGCGATTTCTACTACATGGGGGCGTTCTTCGGGGGGTCGGTGCAAGAGGTGCAGCGGCTCACCAGGGCCTGCCACCAGGCCATGATGGTCGACCAGGCCAACGGCATCGAGGCCGTGTGGCACGACGAGAGCCACCTGAACAAGTACCTACTGCGCCACAAACCCACCAAGGTGCTCTCCCCCGAGTACTTGTGGGACCAGCAGCTGCTGGGCTGGCCCGCCGTCCTGAGGAAGCTGAGGTTCACTGCGGTGCCCAAGAACCACCAGGCGGTCCGGAACCCGTGAGCGGCTGCCAGGGGCTCTGGGAGGGCTGCCGGCAGCCCCGTCCCCCTCCCGCCCTTGGTTTTAGCAGAACGGGTAAACTCTGTTTCCTTTGTCCGTCCTGTTGTGAGTAACTGAAGCCTAGGCCCC |

**Supplemental Table S2** Sequencing primers.

| Samples | Primers | Sequences |
| --- | --- | --- |
| c.98+3 A>G | 98+3-1SF | 5’CCACTGCTTACTGGCTTATCG 3’ |
|  | 98+3-1SR | 5’ CAACAGATGGCTGGCAACTA 3’ |
|  | 98+3-2SF | 5’ CTGTGTCCTGAGTCTTGGC 3’ |
|  | 98+3-2SR | 5’AGCTTCAGGAAAGCCACGTA 3’ |
|  | 98+3-3SF | 5’ATGGCCGAGGTGTTGCGGAC 3’ |
|  | 98+3-3SR | 5’ AGCTTCAGGAAAGCCACGTA 3’ |
| c.155+1G>T | 155+1-1SF | 5’CCACTGCTTACTGGCTTATCG 3’ |
|  | 155+1-1SR | 5’CAACAGATGGCTGGCAACTA 3’ |
|  | 155+1-2SF | 5’GGTCCTAAGCCCCAGAAGTC 3’ |
|  | 155+1-2SR | 5’ AGGTCAAGGCTGACTCCAGA 3’ |
|  | 155+1-3SF | 5’ ATGGCCGAGGTGTTGCGGAC 3’ |
|  | 155+1-3SR | 5’AGCTTCAGGAAAGCCACGTA 3’ |

**Supplementary Table S3** qRT-PCR analysis of mRNA expression for transfected constructs.

| Transfected constructs | Ct(ABO) | Ct(GAPDH) | ΔCt | ΔΔCt | 2^(-ΔΔCt) |
| --- | --- | --- | --- | --- | --- |
| pcDNA3.1(+) | 28.55±1.26 | 14.95±1.31 | 13.6 ± 0.71 | 0 | 1 |
| B101 Control | 27.54±0.5 | 14.12±0.14 | 13.42 ± 0.53 | -0.18±0.53 | 1.22±0.49 |
| B101 del exon 2 | 28.57±1.47 | 14.48±1.04 | 14.09 ± 2.03 | 0.49±2.03 | 1.83±2.16 |
| B101 del exon 3 | 27.07±1.63 | 14.19±0.55 | 12.88 ± 1.09 | -0.72±1.09 | 2.11±1.26 |

Total mRNA levels of the transfected constructs were quantified and normalized to GAPDH. Data are presented as mean ± SD (n=3). *p*=0.8044.

**Supplementary Table S4** GTB activity in supernatants of cells transfected with different cDNA

| cDNA Kinds | GTB Activity | |
| --- | --- | --- |
|  | 4h | 24h |
| *ABO*B.01* wild type cDNA | 1:512* | 1:512* |
| *ABO*B.01* cDNA without exon 2 | - | - |
| *ABO*B.01* cDNA without exon 3 | - | - |
| Empty vector | - | - |

*The maximum dilution ratio in this experiment was 1:512, and no testing was performed at higher dilution ratios.

**Supplementary Figure S1 Dose-response of** **GTB activity in supernatant from cells expressing the wild-type *ABO*B.01* construct**.

This figure plots the agglutination strength against the dilution factor of the anti-B reagent, illustrating the correlation between the antibody dilution (an inverse indicator of detection threshold) and the measurable activity response.


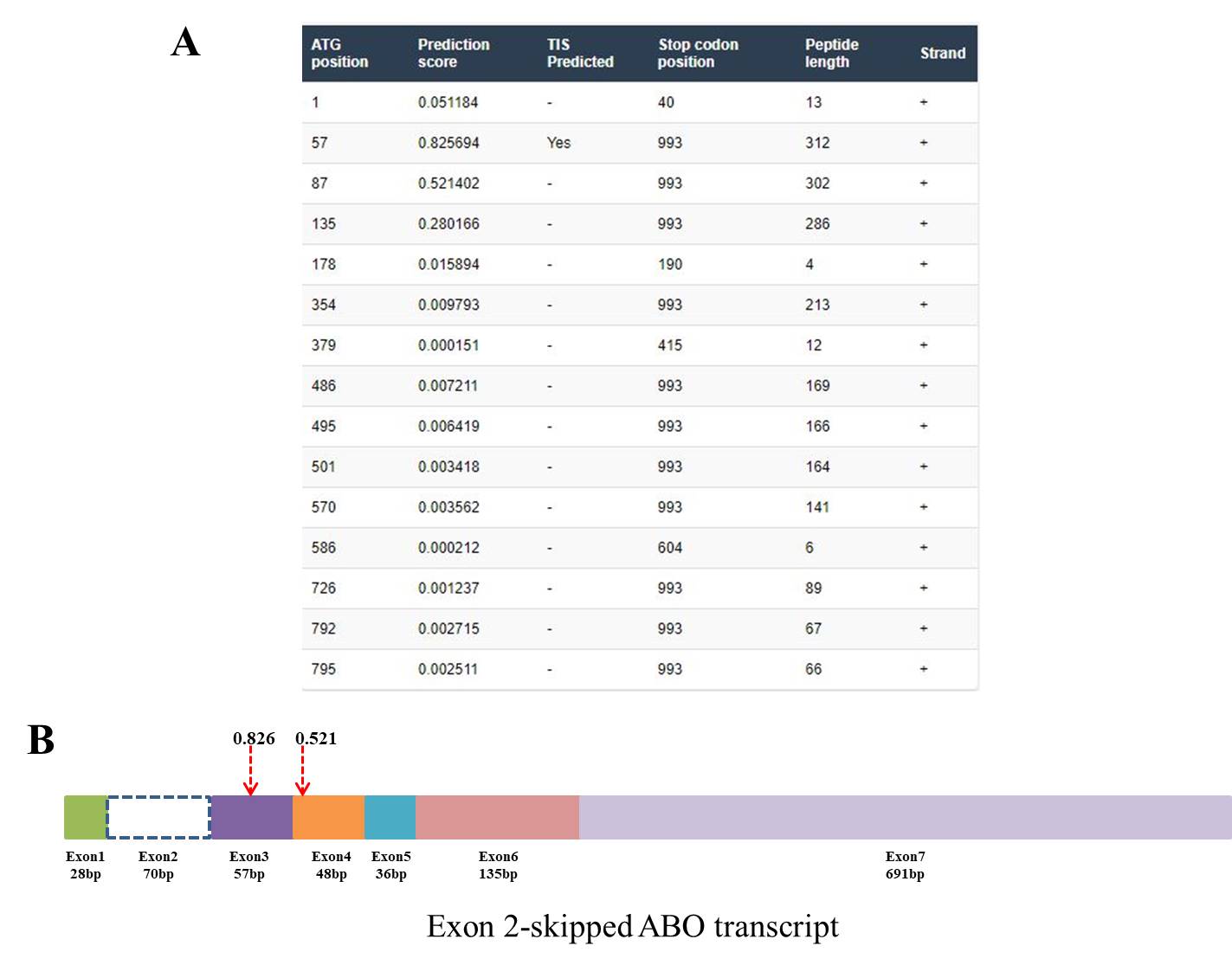


**Supplementary Figure S2 In silico prediction of potential alternative translation initiation sites in the ABO transcript lacking exon 2.**

Computational prediction was performed using NetStart 2.0 to identify potential downstream translation initiation sites in the exon 2‑skipped ABO transcript. (A) Full prediction results from NetStart 2.0. (B) Schematic representation of the prediction outcome. The red arrow points to the potential alternative translation initiation sites (score >0.5).The ATG codon at nucleotide position 57 (located in exon 3) was identified as the most probable downstream translation start site, with a prediction score of 0.826. These predictions indicate a theoretical possibility of alternative translation initiation; however, no functional protein products from such sites were detected in our experimental system, and further investigation is warranted to validate their biological relevance.
